# Use of Homeopathy in Pediatric Oncology in Germany

**DOI:** 10.1155/2011/867151

**Published:** 2010-09-22

**Authors:** Alfred Längler, Claudia Spix, Friedrich Edelhäuser, Genn Kameda, Peter Kaatsch, Georg Seifert

**Affiliations:** ^1^Department of Pediatric and Adolescent Medicine, Gemeinschaftskrankenhaus Herdecke, Gerhard-Kienle-Weg 4, 58313 Herdecke, Germany; ^2^University of Witten/Herdecke, Witten, Germany; ^3^German Childhood Cancer Registry (GCCR), The Institute of Medical Biostatistics, Epidemiology, and Informatics (IMBEI), University of Mainz, Mainz, Germany; ^4^Department of Pediatric Oncology and Hematology, Otto Heubner Centre of Pediatric and Adolescent Medicine, Charité-Universitätsmedizin Berlin, Berlin, Germany

## Abstract

Homeopathy is a frequently used complementary and alternative medicine (CAM) treatment. We present results comparing responses of homeopathy users (HUs) and users of other forms of CAM (NHUs) in pediatric oncology (PO) in Germany. Differences between these two groups (usage, associated demographic characteristics, previous experience with CAM) are investigated. 186 (45.2%) of the 367 CAM users were exposed to homeopathy. The treatment duration amounted to a median of 601 days for HUs and 282 days for NHUs. Parents with p (127; 76.5%) also used homeopathy for their child's cancer. Nonmedical practitioners played a considerably greater role as source of information than did treating physician. In the majority HUs received their prescriptions from nonmedical practitioners (56%; 29.4% of NHUs). HUs communicate more frequently with their physicians about the CAM-use (77.7% versus 65.2%) and recommend CAM more often than NHUs (94% versus 85.6%). Homeopathy is the most frequently used CAM treatment in PO in Germany. HUs sustain treatment and therapies considerably longer than NHUs. Most families who had used homeopathy before their child was diagnosed with cancer also used homeopathy for the treatment of their child's cancer. Compared to other CAM treatments, patient satisfaction with homeopathy appears to be very high.

## 1. Introduction

Complementary and alternative therapies (CAM) are frequently used in the treatment of acute and chronic disease both in Germany and worldwide. This applies equally to adults [1–3] and children [1–4]. Published data on the frequency of CAM used specifically in pediatric oncology is available in the form of usually smaller single-centre studies [5–14]. A parent survey on use of CAM in pediatric oncology published by us involved 1595 parents and is both the most extensive and the only population-based study of this kind in the international literature to date. This survey showed that CAM was used by 35% of the 1063 patients whose parents participated in the study [15]. An exploratory multiple analysis showed that the following factors had a significant influence on the probability of CAM use (in order of importance): earlier experience of CAM (OR = 4.72, *P* < .0001), diagnosis with poor prognosis (OR = 1.63, *P* = .0013), child died before the survey (OR = 1.97, *P* = .0063), and higher social status (OR = 1.44, *P* = .1264). Despite the dispute concerning homoeopathy [16], we assume a growing number of patients using homoeopathy in pediatric oncology. Therefore, it is of interest to examine the use of homoeopathy and user profiles among children with oncological malignancies in Germany.

No studies on the use of homeopathy in pediatric oncology have been published to date. With the exception of a few supportive indications, homeopathic medication in pediatric oncology is used as an adjunct to conventional medicine. Homoeopathy is a well-known system of medicine that follows the “principle of similar” which treats “like with like” with potentized substances at a dilution level far beyond the Avogadro number. The majority of homoeopathic remedies are made from natural substances (e.g., plants, minerals, or animals). Two large and methodologically sophisticated epidemiological studies on the use of homeopathy in children in Germany were recently published [17, 18]. Homeopathy is prescribed both by doctors and by nonmedical practitioners (“Heilpraktiker,” naturopaths, chiropractors, etc.) and also used as self-medication [19, 20]. The publications on the use of CAM in pediatric oncology show consistently that homeopathy plays a significant role in many developed countries as well as in other countries such as India, for example. 

In this retrospective, representative, and population-based parent survey, we compared the group of homeopathy users (Hus) with the users of other CAM therapies (NHUs) to establish whether there are any differences between these two groups with regard to patterns of CAM-use, the attending circumstances, or previous experience of CAM.

## 2. Patients and Methods

The postal survey sent to parents was carried out in 2004 in collaboration with the Deutsche Kinderkrebsregister (GCCR) (German Childhood Cancer Registry). The study population included all parents in Germany with a child (under the age of 15 years) diagnosed in 2001 with one of the diseases registered and systematically recorded by the GCCR. At least 95% of all German cases of childhood cancer are registered in the GCCR. Exclusion criteria were death within the first 8 weeks after diagnosis and development of a second cancer. The survey was conducted in coordination with all German hospitals that had treated children with leukemia and cancer in the year 2001 and had reported to the GCCR. The hospitals were permitted to exclude individual patients from the survey (stating reasons if possible). The selected families were sent the questionnaire by mail. The list of alternative and complementary treatment methods given in the questionnaire was as comprehensive as possible in order to obtain as realistic as possible a picture of the different methods used. The systematically developed German language questionnaire for parents was based on data published on this topic to date, our own clinical experience, and the experience obtained from a pilot survey [21].

Among other things, the questionnaire provided an alphabetic list of 69 possible CAM treatments and therapies, of which “homeopathy” was one. Those CAM-users who named this category were defined as homeopathy users (Hus) and their data compared with those of the group of nonhomeopathy users (NHUs) (i.e., users of other forms of complementary or alternative medicine but not homeopathy) and examined with respect to patterns of CAM-use, attending circumstances, and previous experience of CAM.

All patients had received conventional treatment as well as the specified complementary treatments or therapies. Because we had no information from so-called “treatment refusers,” we are unable to comment on their use of complementary and alternative therapies. But “treatment refusers” are extremely rare in Germany. The study was approved by the ethics committee of the University of Witten/Herdecke, Germany, and carried out in accordance with the World Medical Association Declaration of Helsinki [22].

## 3. Statistical Analysis

This was not an analytical study and therefore results are presented primarily in the form of descriptive statistics, that is, percentages relating to the mostly categorical data collected. The basic percentages are supplemented by binomial 95% confidence intervals (95% CI) reflecting the precision of the estimate. The main measure is the comparison of percentages between the groups. Some of the more relevant differences between the HU and NHU groups were tested by the *χ* ^2^-test for homogeneity in tables. These tests should also be viewed as exploratory in nature, not confirmatory.

## 4. Results

### 4.1. CAM Use

Of the 1063 families who responded, 367 (35%, 95% CI (31.7%; 37.4%)) stated that they had used CAM in the course of their child's illness. Of these 367 CAM users, 166 (45.2%) reported that they had used homeopathic medicines. This was thus the numerically largest group of CAM users. When asked for the “most important” CAM therapies used, 137 (37.3%) of all CAM-users reported that they had given homeopathic medicines (Table 1).

The mean duration of use of homeopathic medicines was 601 days. The mean duration of use of all other CAM therapies was 282 days. These are lowest estimates, as in many cases, treatments were still ongoing at the time of the survey. 

An analysis of the frequency of use of different CAM-therapies by diagnostic group on the basis of the diagnostic groups recorded in the GCCR did not show any particular concentration of homeopathy users in specific diagnostic groups (data not shown). A total of 396 (37.3%) of the survey participants had previous experience of CAM. This previous experience was most often with homeopathy (*n* = 280; multiple responses allowed). Of the 166 homeopathy users 127 (76.5%) had previous experience of homeopathy. A further 38 (22.9%) homeopathy users had no previous CAM experience. In the NHU group (*n* = 201) on the other hand, none of the respondents had previous experience of homeopathy, 100 (49.8%) had previous experience of other CAM therapies and 98 (48.8%) had no previous CAM experience (not specified 3 (1.5%)) (Table 2). Comparison of these two groups with regard to their previous experience of homeopathy shows a highly significant difference (*P* < .0001).

There was no difference between the HU and NHU groups with regard to either the prognosis of the underlying disease or the attribute “death of the child before participation” (data not shown). The percentage of HUs with high social status was the same as in the group of NHUs (52% and 47.6%, resp.). 

### 4.2. Attending Circumstances of CAM Use

Comparison between HUs and NHUs with regard to the reasons for using CAM showed that the categories “for physical stabilization” (77.7% versus 63.2%), “to enhance the immune system” (72.3% versus 61.2%), “to improve the tolerability of the conventional treatment” (58.4% versus 39.3%), and “for detoxification” (42.2% versus 19.9%) were named noticeably more often by HUs. The only category named more often by NHUs than HUs was “for relaxation” (21.4% versus 12.7%). 

**Figure 1:**
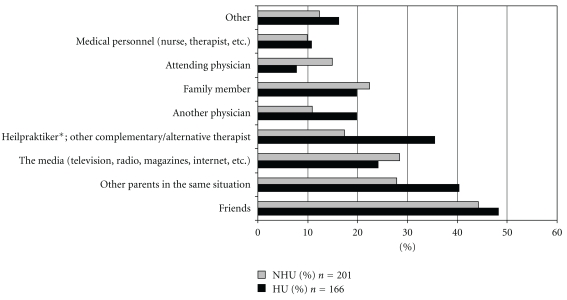
Sources of information about CAM (*n* = 367 CAM-users; multiple answers possible). *Heilpraktiker: state registered, non-physician health care provider of CAM in Germany, Austria and Switzerland.

**Figure 2:**
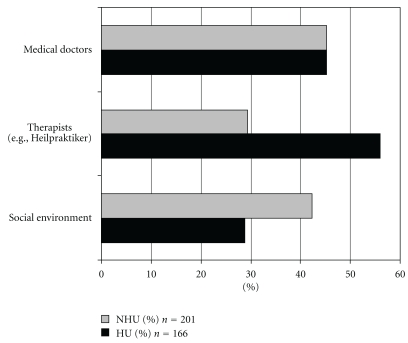
CAM-prescribers (*n* = 367 CAM-users; multiple answers possible).

With regard to the sources of information on CAM the survey showed that for the HUs nonmedical practitioners played a considerably greater role than treating physicians (Figure 1). In the large majority of cases HUs received their prescriptions from nonmedical practitioners (56% versus 29.4% of NHUs). Self-medication played a lesser role for HUs than NHUs (13.8% versus 23.4%). The same holds for the social environment (28.9% versus 42.3%) (Figure 2).

The timing of CAM use in the course of the illness showed about the same distribution in both groups. In most cases CAM was used at the same time as the conventional treatment performed by the pediatric oncologist. 14% of the users had used, CAM only after the end of the conventional therapy.

### 4.3. Patterns of Communication

The percentage of families who spoke with their doctors about using CAM with their child was particularly high amongst the HUs. A total of 77.7% of the HUs spoke to a doctor about use of CAM compared with about two thirds of the NHUs (65.2%).

With regard to the doctors' responses (“recommended,” “took note,” “advised against,” “do not know”) there were significant differences between the hospital doctors addressed by HUs and NHUs. 7.3% of the HU group reported that the hospital doctors approached by them had recommended use compared with 17.3% of the NHU group (*P* = .02). 72.7% of the hospital doctors of the HUs took note of the CAM use without comment compared with 55.5% of the doctors of NHUs while 20% of the hospital doctors of HUs advised against CAM use compared with 27.3% of doctors of NHUs. There was no identifiable difference between HUs and NHUs with regard to the reactions of the pediatricians or general practitioners addressed.

### 4.4. Hoped for and Experienced Effect

There was no essential difference between the two groups with regard to their basic conviction about the efficacy of CAM; before use 68% of the HUs and 59.2% of the NHUs were “absolutely sure” or “fairly sure” that CAM would have a positive impact on their child's illness. However, the percentage of “doubters” was higher in the NHU group (31.8% versus 21.1%). In the NHU group, the parents' assessments of the actual effect of homeopathy on their child's illness showed no essential differences compared with their expectations (data not shown).

When asked about side-effects, 4.4% of all CAM users reported one nonspecific side-effect (*n* = 16 of 358 of CAM users answering this question, multiple responses were allowed). There were no significant differences between HUs and NHUs (data not shown).

The fundamentally positive attitude (hoped for and experienced effect) of all CAM users is reflected in the willingness of the parents to recommend CAM use to other parents in a similar situation: 89.4% of all CAM users would do this (94% of HUs, 85.6% of NHUs). It is not surprising that HUs would recommend homeopathy most often whereas in the case of NHUs homeopathy is only in 32nd place amongst the recommended CAM therapies. Mistletoe therapy, on the other hand, is frequently recommended in both groups (place 2 for HUs and place 1 for NHUs).

## 5. Discussion

These data are from the most extensive as well as the first population-based study on the prevalence of CAM use in pediatric oncology. There have been no other studies to date either on the use of homeopathy in pediatric oncology or in comparison with users of other CAM therapies. In our survey, population users of homeopathy were the largest group (45.2%). In the results of non-European studies, homeopathy only plays a marginal role if any: Israel 16.4% [23], Canada 1.2% [12], and USA not mentioned [15]. Only one recent study from Italy [24] and a small study from the Netherlands [9] also found that homeopathy was the most frequently mentioned CAM therapy amongst children with cancer. This high rate of homeopathy use confirms the fact that homeopathy in children is used most often for treatment of chronic illnesses or of acute self-limiting illnesses [25–27]. 

An important factor which influences the likelihood of CAM use in a child with cancer is previous experience of CAM in the family before the child's cancer. 37.3% of all survey participants had already had experience of CAM, a figure which is lower than that in the German population as a whole [28, 29]. On the other hand, 76.5% of HUs had previous experience of CAM, always of homeopathy. In comparison with NHUs (27.4% previous experience of homeopathy) this difference is statistically significant and was not previously known.

In HUs more than in NHUs the reasons given for the use of CAM were related to reduction of concomitant symptoms of the illness or of the conventional therapy. But general treatment goals such as “to enhance the immune system” or “for detoxification” were also named disproportionately often by HUs. There are no comparable data in the literature.

A large percentage of HUs (77.7%) had spoken with a doctor (GP, pediatrician, pediatric oncologist) about the use of homeopathic medicines. Surveys on the use of homeopathy in general pediatrics have shown considerably less willingness to talk to a doctor about the homeopathic treatment [25]. But 65.2% of the NHUs in our survey population also spoke to a doctor about their use of CAM therapies. The reactions of the pediatric oncologists approached differed substantially between the two groups, those approached by the NHUs actively recommending CAM use significantly less often (7.3% versus 17.3%).

Although the HUs spoke strikingly often to the doctors involved in their child's treatment, these play only a subordinate role when it comes to the prescribing of the homeopathic medicines. Most prescriptions for homeopathic medicines (which are usually available over-the-counter) came from nonmedical practitioners. This is in agreement with the results of other studies investigating the use of homeopathy in pediatrics [24, 25, 30].

In contrast to an epidemiological study on the status of homeopathy in children in Germany [31], our data show no dependence of the use of homeopathy compared to NHUs on higher social status. However, in the analysis of the total study population (HUs + NHUs) a higher social status was one of the statistically most striking factors influencing the use of CAM in general.

In view of the fundamentally positive expectations of the CAM users, the high percentage who would further recommend CAM to parents in a comparable situation is not surprising. However, here too there is a difference between the two groups, 94% of the HUs stating that they would further recommend CAM compared with 85.6% of the NHUs. Our data also show that HUs continue homeopathic treatment significantly longer than patients using other CAM therapies. A possible reason for the high degree of satisfaction of HUs could be the fact that homeopaths devote more time and attention to their patients. A homeopathic interview can take as long as 1-2 hours. In a qualitative comparative study of homeopaths and conventional doctors it was found that the parents experienced the treatment by a homeopath as more holistically oriented compared with the symptom-based approach of a conventional doctor [32]. This probably also applies to the HUs in this survey, even though the prescribers were for the most part nonmedical practitioners. The data obtained in this survey do not permit any conclusions regarding the effectiveness of the treatment, the occurrence of side-effects, or interactions with the homeopathic medicines. Furthermore, in a double-blind clinical trial published by Paris et al., patients in both groups (active treatment and placebo) were convinced of the effectiveness of homeopathy both before and after the treatment [29]. Nevertheless, the analysis demonstrated the homeopathic treatment to be as ineffective as the placebo. On the one hand, trials of homeopathy trials, as of some other treatments, are associated with high patient conviction of efficacy from the outset. This seems to be a persistent problem and has to be taken into account methodologically. On the other hand, assuming the therapy was effective, the subjective assessment by the parents displays that the complementary homeopathic therapy was associated with a perceived benefit. Whether this had anything to do with an actual therapeutic effect or any other psychological effects, for example, “meaning response” [26] or placebo-effect [32], is not yet known as the methods used in this study do not permit differentiation of these effects. 

## 6. Limitations

As a relatively high percentage of the responding families used anthroposophic medicine (AM) in addition to the homeopathy, the frequency of use of homeopathy in pediatric oncology in Germany may be overreported in this study. Lay people in particular often find it difficult to distinguish between AM and homeopathy as both treatment systems use potentized medicines. It is possible that some of the families who were prescribed medicines by an anthroposophic doctor incorrectly reported this as homeopathy. However, it is unlikely that homeopathic medicines were incorrectly reported as AM. Anthroposophic medicine is partly used as an extension to conventional medicine and partly replaces it [33]. The treatment and therapeutic possibilities offered by AM include specially produced medicines of mineral, plant, and animal origin, various artistic therapies, rhythmic massage [33], curative eurythmy [34], external treatments (compresses, oils and ointments, baths), medical consultations and counselling (partly psychotherapeutic), and extended anthroposophic nursing and care. These treatments and therapies aim to stimulate and strengthen the patient's own healing forces; they are practiced by physicians, therapists, and nurses. Physicians are trained in both AM and conventional medicine and most are medical specialists. A further limitation is that the questionnaire was only available in German and that certain patient groups may therefore have been indirectly excluded from the survey.

## 7. Conclusion

Homeopathy is the most frequently used complementary therapy in pediatric oncology in Germany. Most HUs had used homeopathy before the cancer and would further recommend homeopathy to others in a similar situation.

## Figures and Tables

**Table 1:** The “most important” CAM treatment methods from the users' viewpoint. Selection: treatment methods listed at least 10 times (*n* = 367 CAM-users; multiple answers possible).

CAM treatment methods	Number of patients (%)
Homeopathy	137 (37.3)
Mistletoe therapy	53 (14.4)
Anthroposophic-homeopathic medications (except for mistletoe therapy)	46 (12.5)
Food supplements	43 (11.7)
Reiki	27 (7.4)
Dietary changes	26 (7.1)
Laying on of hands	22 (6.0)
Medicines of plant origin (Phytotherapy)	21 (5.7)
Selenium	21 (5.7)
Vitamin C	21 (5.7)
Massage	19 (5.2)
Other	19 (5.2)
Spiritual healer	18 (4.9)
Ayurveda, for example, H15 (incense)	16 (4.4)
High dosage vitamins	16 (4.4)
Bach Flower Remedies	15 (4.1)
Acupuncture	14 (3.8)
Bioresonance	13 (3.5)
Kinesiology	13 (3.5)
Osteopathy	12 (3.3)
“Biochemistry according to Schüssler”	11 (3.0)
“Energy work”	11 (3.0)
Music therapy	11 (3.0)
…	…

**Table 2:** Previous experience of CAM in the family before a child was diagnosed with cancer (*n* = 1063 questionnaire participants).

Previous experience	CAM-users (*n* = 367)		
	HU (%) *n* = 166	NHU (%) *n* = 201	CAM-nonusers (%) *n* = 696
Previous experience with homeopathy	121 (72,9)	55 (27,4)	104 (14,9)
Previous experience with CAM, but no previous experience with homeopathy	6 (3,6)	45 (22,4)	65 (9,3)
No previous experience of CAM	38 (22,9)	98 (48,8)	517 (74,3)
No answer given	1 (0,6)	3 (1,5)	10 (1,4)
	*P* < .0001		

## References

[1] Cottencin A, Mullet E, Sorum PC (2006). Consulting a complementary and alternative medical practitioner: a systematic inventory of motives among French patients. *Journal of Alternative and Complementary Medicine*.

[2] Ernst E Prevalence of use of complementary/alternative medicine: a systematic review. http://whqlibdoc.who.int/bulletin/2000/Vol78-No2/bulletin_2000_78(2)_252-257.pdf.

[3] Allensbacher Archiv (2002). *Naturheilmittel 2002*.

[4] Fletcher PC, Clarke J (2004). The use of complementary and alternative medicine among pediatric patients. *Cancer Nursing*.

[5] McCann LJ, Newell SJ (2006). Survey of paediatric complementary and alternative medicine use in health and chronic illness. *Archives of Disease in Childhood*.

[6] Shaw A, Thompson EA, Sharp D (2006). Complementary therapy use by patients and parents of children with asthma and the implications for NHS care: a qualitative study. *BMC Health Services Research*.

[7] Zuzak TJ, Zuzak-Siegrist I, Simões-Wüst AP, Rist L, Staubli G (2009). Use of complementary and alternative medicine by patients presenting to a paediatric Emergency Department. *European Journal of Pediatrics*.

[8] Molassiotis A, Cubbin D (2004). ’Thinking outside the box’: complementary and alternative therapies use in paediatric oncology patients. *European Journal of Oncology Nursing*.

[9] Grootenhuis MA, Last BF, de Graaf-Nijkerk JH, van der Wel M (1998). Use of alternative treatment in pediatric oncology. *Cancer Nursing*.

[10] Mottonen M, Uhari M (1997). Use of micronutrients and alternative drugs by children with acute lymphoblastic leukemia. *Medical and Pediatric Oncology*.

[11] Bold J, Leis A (2001). Unconventional therapy use among children with cancer in Saskatchewan. *Journal of Pediatric Oncology Nursing*.

[12] Fernandez CV, Stutzer CA, MacWilliam L, Fryer C (1998). Alternative and complementary therapy use in pediatric oncology patients in British Columbia: prevalence and reasons for use and nonuse. *Journal of Clinical Oncology*.

[13] Friedman T, Slayton WB, Allen LS (1997). Use of alternative therapies for children with cancer. *Pediatrics*.

[14] Martel D, Bussières J-F, Théorêt Y (2005). Use of alternative and complementary therapies in children with cancer. *Pediatric Blood and Cancer*.

[15] Neuhouser ML, Patterson RE, Schwartz SM, Hedderson MM, Bowen DJ, Standish LJ (2001). Use of alternative medicine by children with cancer in Washington State. *Preventive Medicine*.

[16] Ben Arush MW, Geva H, Ofir R, Mashiach T, Uziel R, Dashkovsky Z (2006). Prevalence and characteristics of complementary medicine used by pediatric cancer patients in a mixed western and middle-eastern population. *Journal of Pediatric Hematology/Oncology*.

[17] Yeh C-H, Tsai J-L, Li W (2000). Use of alternative therapy among pediatric oncology patients in Taiwan. *Pediatric Hematology and Oncology*.

[18] Zutavern A, Rzehak P, Brockow I (2007). Day care in relation to respiratory-tract and gastrointestinal infections in a German birth cohort study. *Acta Paediatrica*.

[19] Laengler A, Spix C, Seifert G, Gottschling S, Graf N, Kaatsch P (2008). Complementary and alternative treatment methods in children with cancer: a population-based retrospective survey on the prevalence of use in Germany. *European Journal of Cancer*.

[20] Fisher P (2006). Homeopathy and the lancet. *Evidence-Based Complementary and Alternative Medicine*.

[21] Längler A, Spix C, Gottschling S, Graf N, Kaatsch P (2005). Elternbefragung zur Anwendung alternativer und komplementärer Behandlungsmethoden in der Kinderonkologie in Deutschland. *Klinische Padiatrie*.

[22] http://www.wma.net/e/policy/pdf/17c.pdf.

[23] Ben Arush MW, Geva H, Ofir R, Mashiach T, Uziel R, Dashkovsky Z (2006). Prevalence and characteristics of complementary medicine used by pediatric cancer patients in a mixed western and middle-eastern population. *Journal of Pediatric Hematology/Oncology*.

[24] Steinsbekk A, Bentzen N, Brien S (2006). Why do parents take their children to homeopaths? — an exploratory qualitative study. *Forschende Komplementärmedizin*.

[25] Ekins-Daukes S, Helms PJ, Taylor MW, Simpson CR, McLay JS (2005). Paediatric homoeopathy in general practice: where, when and why?. *British Journal of Clinical Pharmacology*.

[26] Huber R, Koch D, Beisner I, Zschocke I, Ludtke R (2004). Experience and attitudes towards CAM—a survey of internal and psychosomatic patients in a German University Hospital. *Alternative Therapies in Health and Medicine*.

[27] Härtel U, Volger E (2004). Use and acceptance of classical natural and alternative medicine in Germany—findings of a representative population-based survey. *Forschende Komplementarmedizin und Klassische Naturheilkunde*.

[28] Marian F, Joost K, Saini KD, von Ammon K, Thurneysen A, Busato A (2008). Patient satisfaction and side effects in primary care: an observational study comparing homeopathy and conventional medicine. *BMC Complementary and Alternative Medicine*.

[29] Paris A, Gonnet N, Chaussard C (2008). Effect of homeopathy on analgesic intake following knee ligament reconstruction: a phase III monocentre randomized placebo controlled study. *British Journal of Clinical Pharmacology*.

[30] Lee ACC, Kemper KJ (2000). Homeopathy and naturopathy: practice characteristics and pediatric care. *Archives of Pediatrics and Adolescent Medicine*.

[31] Du Y, Knopf H (2009). Paediatric homoeopathy in Germany: results of the German Health Interview and Examination Survey for Children and Adolescents (KiGGS). *Pharmacoepidemiology and Drug Safety*.

[32] Moerman DE, Jonas WB (2002). Deconstructing the placebo effect and finding the meaning response. *Annals of Internal Medicine*.

[33] http://www.ivaa.info/IVAA_new/factsheet.htm.

[34] Büssing A, Ostermann T, Majorek M, Matthiessen PF (2008). Eurythmy therapy in clinical studies: a systematic literature review. *BMC Complementary and Alternative Medicine*.

